# Stem cell therapy: a revolutionary cure or a pandora's box

**DOI:** 10.1186/s13287-025-04334-1

**Published:** 2025-05-22

**Authors:** Hany E. Marei

**Affiliations:** Department of Cytology and Histology, Faculty of Veterinary Medicine, Mansoura University, Mansoura, 35116 Egypt

**Keywords:** Stem cell research, Regenerative medicine, Induced pluripotent stem cells (iPSCs), Embryonic stem cells (ESCs), Mesenchymal stem cells (MSCs), Hematopoietic stem cells (HSCs), Organoids, Disease modeling, Gene editing, CRISPR-Cas9, Neurodegenerative disorders, Alzheimer’s disease, Parkinson’s disease, Spinal cord injury, Diabetes treatment, Cardiovascular regeneration, Tissue engineering, Organ transplantation, Ethical concerns, Regulatory challenges, Commercialization, Biotech investments, Unregulated stem cell clinics, Reproducibility issues, Clinical applications, Tumor formation risk, Immune rejection, Standardization of protocols, Future of stem cell therapies

## Abstract

This review article examines how stem cell therapies can cure various diseases and injuries while also discussing the difficulties and moral conundrums that come with their application. The article focuses on the revolutionary developments in stem cell research, especially the introduction of gene editing tools like CRISPR-Cas9, which can potentially improve the safety and effectiveness of stem cell-based treatments. To guarantee the responsible use of stem cells in clinical applications, it is also argued that standardizing clinical procedures and fortifying ethical and regulatory frameworks are essential first steps. The assessment also highlights the substantial obstacles that still need to be addressed, such as the moral dilemmas raised by the use of embryonic stem cells, the dangers of unlicensed stem cell clinics, and the difficulties in obtaining and paying for care for patients. The study emphasizes how critical it is to address these problems to stop exploitation, guarantee patient safety, and increase the accessibility of stem cell therapy. The review also addresses the significance of thorough clinical trials, public education, and policy development to guarantee that stem cell research may fulfill its full potential. The review concludes by describing stem cell research as a promising but complicated topic that necessitates a thorough evaluation of both the hazards and the benefits. To overcome the ethical, legal, and accessibility obstacles and eventually guarantee that stem cell treatments may be safely and fairly included in conventional healthcare, it urges cooperation between the scientific community, legislators, and the general public.

## Introduction

Stem cells are essential to developmental biology and regenerative medicine because they are undifferentiated cells with the remarkable capacity to renew and differentiate into specialized cell types. The idea was born when German biologist Ernst Haeckel initially proposed the concept of stem cells in the late nineteenth century. However, the discovery of hematopoietic stem cells (HSCs) by Till and McCulloch in 1961 marked the beginning of the contemporary age of stem cell research. Thomson et al. isolated human embryonic stem cells (ESCs) in 1998. These ground-breaking findings sparked optimism about using stem cells to replace damaged or defective cells in treating various illnesses, such as diabetes, cardiovascular disease, and neurodegenerative disorders. By allowing patient-specific cell therapies without the ethical issues surrounding ESCs, Takahashi and Yamanaka's discovery of induced pluripotent stem cells (iPSCs) in 2006 considerably broadened the therapeutic landscape. The safety and feasibility of stem cell-based therapies have been doubted despite these guarantees because of significant problems such as immunological rejection, tumorigenicity, and ethical dilemmas [1–3].

Although stem cell research has seen many in vitro and preclinical success stories, human clinical outcomes have been less than ideal. Many businesses and technological companies in the stem cell industry failed to produce a convincing, reproducible, FDA-licensed, and effective stem cell remedy, undermining the excellent hope for desperate patients searching for a genuine solution for many diseases with poor prognoses and incurable conditions. The current reality of stem cell research is that it is ongoing at full capacity. Still, the promising rewards and drastic outcomes for a real cure for most stem-cell-directed cures have not been met in most cases yet. According to the current state of stem cell research, although the field is working at full capacity, the dramatic results and promising rewards for proper treatment for most stem cell-directed diseases have not yet been realized. At the same time, more commercials and promises of stem cell potential exist everywhere. Patients' confidence and regulatory agencies, which apply stringent standard criteria before clinical approval, have suffered dramatically due to this circumstance.

Despite all of these uncertainties, stem cell research still receives a large amount of funding from major funding agencies, and stem cell research experts are working harder to demonstrate that their more than 60 years of efforts are still necessary and that their work is noteworthy and promising given the enormous potential of future stem cell research.

Difficulties in converting preclinical achievements into clinical results are not specific to stem cell therapies but are typical of many different fields of pharmaceutical research. Statistical data on the low success rates of Phase III clinical trials in the pharmaceutical sector, where around 90% of trials fail to produce a new treatment reaching the market [4]. This analogy currently emphasizes that stem cell therapies encounter the same challenges as other forms of medical treatment, including problems with clinical efficacy, safety concerns, and regulatory approval.

While limbal stem cell transplantation has become a conventional treatment for recovering eyesight in people with some kinds of blindness, HSCT is a standard therapeutic option that saves thousands of lives yearly, especially in patients with hematologic tumors. Moreover, several individuals in recent clinical studies, including iPSC-derived insulin-producing pancreatic beta cells, stayed off insulin for over a year and exhibited encouraging outcomes [5–7].

In addition to highlighting preclinical and clinical success stories, this review seeks to examine the true potential of stem cell research critically and offer a thorough analysis of how experts in the field are considering their next course of action and how they plan to overcome significant obstacles that still stand in the way of the complete application of stem cell research to provide a proper regenerative solution to central stem cell-targeted diseases, such as cardiovascular, neurodegenerative, diabetes, genetic diseases, and more.

## Major advances in stem cell research

Thanks to technological advancements and a better comprehension of cellular biology, stem cell research has advanced significantly over the last ten years. Researchers have improved their medicinal use, addressed safety problems, and increased their capacity to manipulate stem cells. In addition to increasing the possibility of stem cell-based treatments, these developments have brought up new moral and scientific issues. This section lists recent discoveries that have influenced the field, including induced pluripotent stem cells (iPSCs), organoids and disease Modeling, gene editing and CRISPR-Cas9, and clinical applications.

### Induced pluripotent stem cells (iPSCs)

iPSCs have transformed stem cell research and regenerative medicine as a flexible and moral substitute for embryonic stem cells (ESCs). Since their development, iPSCs have demonstrated enormous promise in drug discovery, disease modeling, and possible clinical uses. However, several issues still hampered their complete therapeutic implementation.

**Fig. 1:**
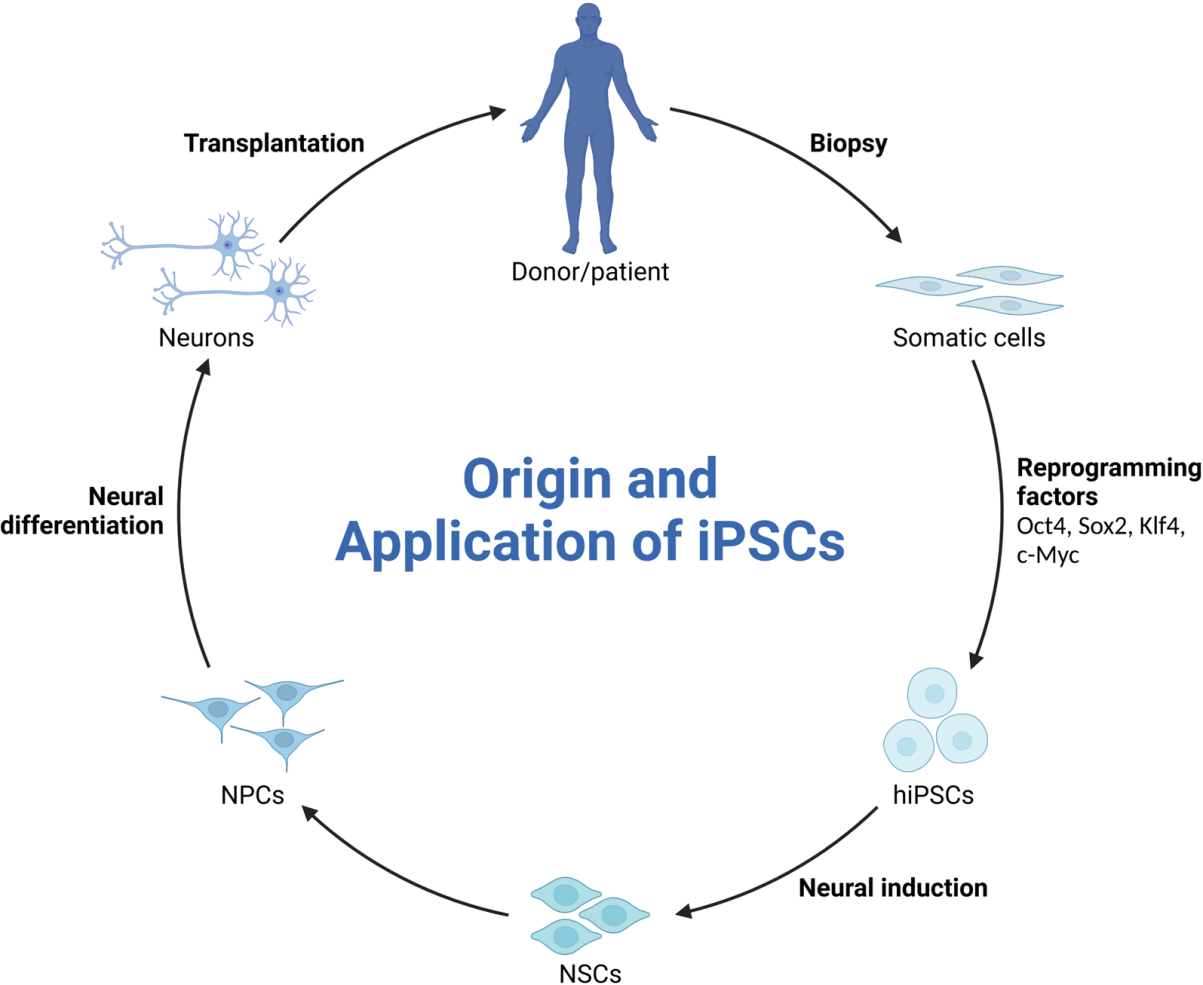
Origin and application of iPSCs. This figure shows how essential transcription factors can be added to reprogramming somatic cells into induced pluripotent stem cells (iPSCs). It demonstrates the potential of iPSCs for patient-specific therapy, personalized medicine, and the investigation of genetic abnormalities, as well as their adaptability in regenerative medicine, disease modeling, and drug development. The neural induction and differentiation of iPSCs into neural stem cells (NSCs), neural progenitor cells (NPCs), and neuronal differentiation into neurons employed in vitro studies and transplantation are also depicted in the figure

Adult cells are reprogrammed into an embryonic-like pluripotent state by introducing transcription factors known as iPSCs. Shinya Yamanaka and Kazutoshi Takahashi initially made this revolutionary finding in 2006 when they successfully reprogrammed mouse fibroblasts using four essential transcription factors: Oct3/4, Sox2, Klf4, and c-Myc. A significant turning point in stem cell research was reached in 2007 when the method was modified for use with human cells [2] (Fig. 1).

While iPSCs and ESCs have many things in common, they differ significantly. iPSCs can differentiate into any cell, just like ESCs. However, unlike ESCs produced from embryos, iPSCs do not have the same ethical issues. iPSCs occasionally display epigenetic memory from their initial somatic state, but ESCs are considered the gold standard for pluripotency. Since iPSCs come from somatic cells, they avoid moral questions about embryo annihilation, a major ethical issue connected with hESCs. Since it usually destroys viable embryos, the procurement of embryos for hESC formation raises serious ethical problems. Nevertheless, most hESCs employed in research today come from discarded embryos produced by assisted reproduction; otherwise, these would be destroyed if not used for stem cell research. This supply of embryos lessens the ethical questions about hESCs, and the increasing usage of iPSCs marks a change from these conundrums. Emphasizing the growing reliance on iPSCs as a more morally acceptable option [8].

iPSCs can differentiate into any cell, unlike adult stem cells (ASCs), which are usually multipotent and have a limited capacity for differentiation. However, ASCs are safer in some therapeutic applications because they do not require genetic alteration to function. Because of their ability to differentiate into mesodermal lineages and their immunomodulatory capabilities, mesenchymal stem cells (MSCs) are used extensively. However, compared to iPSCs, their plasticity is substantially less.

The many benefits of iPSCs have led to their extensive application in scientific research. Since they do not necessitate the destruction of embryos, they are morally acceptable. Additionally, they make patient-specific therapy possible, which lowers the possibility of immunological rejection in prospective therapies. Furthermore, they offer an endless supply because iPSCs may be produced from readily available cells such as skin fibroblasts or blood cells. Additionally, by developing patient-specific illness models in vitro, iPSCs have improved disease modeling capabilities, enabling researchers to investigate hereditary abnormalities. Additionally, they make drug screening easier, allowing more precise drug testing and toxicity evaluation in models that resemble humans.

Despite its potential, several challenges must be overcome before iPSCs can be widely used in clinical settings. Genetic alterations may be introduced during the reprogramming process, raising the possibility of tumorigenicity. The ability of certain iPSCs to differentiate is impacted by the retention of memory of their original tissue. iPSC generation is still an inefficient procedure that has to be optimized. Tumor formation is increased when oncogenic transcription factors, such as c-Myc, are used. Furthermore, it is still expensive to produce iPSCs on a big scale and translate them into therapeutic settings [9].

Numerous innovations have shown how promising iPSCs can be. Cardiomyocytes produced from iPSCs have been utilized to study heart problems and provide medication therapies for ailments, including hypertrophic cardiomyopathy. iPSC models have been instrumental in studying Parkinson’s and Alzheimer’s disease, enabling the screening of neuroprotective drugs. Blood cells from iPSCs have been investigated, and treatments for leukemia and sickle cell anemia are possible. Treatment for macular degeneration with iPSC-derived retinal pigment epithelial cells has shown promise in clinical trials [8].

Several new paths must be pursued to improve the therapeutic application of iPSCs and overcome current obstacles. Improving reprogramming processes by developing safer and more effective solutions, like non-integrational ways, is crucial. Another goal is to create standardized techniques and enhance differentiation regimens to produce functionally mature cells. The therapeutic potential of iPSCs will be further increased by combining them with CRISPR-Cas9 to fix mutations that cause disease. Safety will be improved by addressing tumorigenicity with small medicines or gene-editing techniques. Finally, more clinical trials and regulatory approval are required to confirm the safety and effectiveness of iPSC-based treatments [10].

iPSCs have emerged as a transformative tool in biomedical research, offering significant potential in personalized medicine, disease modeling, and regenerative therapies. While several challenges remain, advancements in reprogramming techniques, genome editing, and safety measures continue refining the field. By addressing current limitations, iPSCs may soon fulfill their promise as a viable alternative to traditional stem cell therapies, which will pave the way for groundbreaking clinical applications. In biomedical research, pluripotent stem cells have become a game-changing technique with great promise for regenerative therapies, disease models, and personalized medicine. Although there are still several obstacles to overcome, the field is being improved by continuous developments in genome editing, reprogramming methods, and safety precautions. By overcoming present obstacles, iPSCs could soon live up to their potential as a competitive substitute for conventional stem cell treatments, opening the door for ground-breaking therapeutic uses shortly.

### Organoids and disease modeling

Although iPSCs have greatly improved our capacity to develop regenerative treatments and model illnesses, their full potential is frequently achieved with three-dimensional (3D) culture platforms like organoids. Organoids are multicellular, self-organizing entities from stem cells that offer a more physiologically appropriate platform for research on medication reactions, disease progression, and tissue formation. Researchers can more precisely replicate the complexity of human organs in vitro by using iPSCs to develop patient-specific organoids. By combining iPSCs with organoid technology, new approaches to disease modeling have been made possible, providing previously unheard-of insights into ailments ranging from cancer to neurodegenerative diseases.

**Fig. 2:**
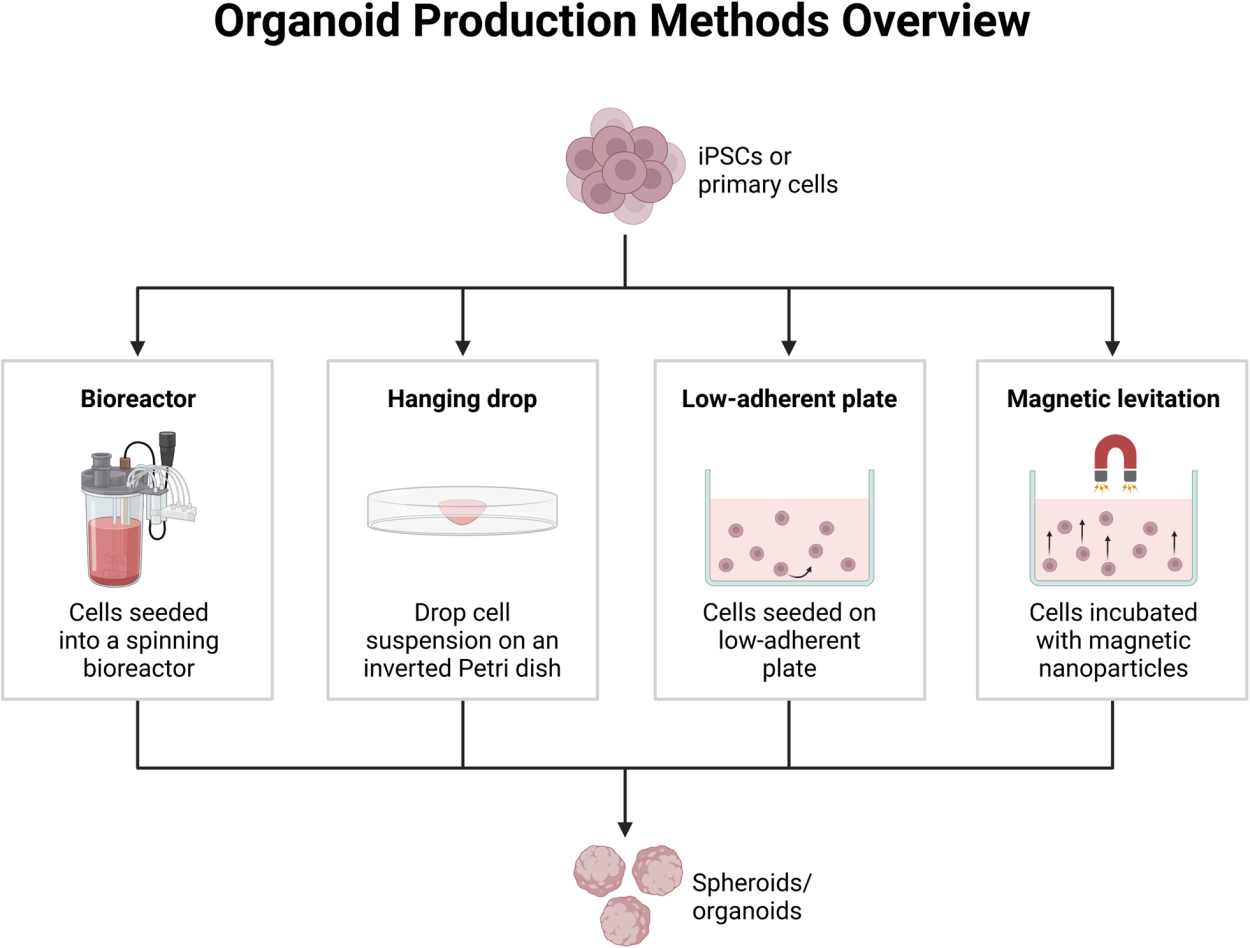
Organoid production methods overview. This figure shows several techniques for developing organoids from stem cells, including growth factor signaling, bioreactors, and extracellular matrices. It illustrates the several stages of organoid growth, from early stem cell culture to three-dimensional self-organization into tissue-like structures. The employment of organoids in disease-cause research, drug candidate screening, and organ development modeling exemplifies their importance in translational research

In biomedical research, organoids have become a game-changing tool that bridges the gap between vivo models and conventional two-dimensional (2D) cell cultures (Fig. 2). Researchers successfully generated self-organizing intestinal structures from adult stem cells in the early 2010s, leading to the development of the first organoids [11]. Since then, models of the brain, liver, kidneys, pancreas, and other tissues have been added to the repertoire of organoid technology, offering a previously unheard-of platform for researching medication responses, disease causes, and organ development.

Organoids are widely used in disease modeling, where they are used as physiologically relevant systems to investigate disorders like infectious diseases, cancer, and neurological diseases [12]. Organoids produced by patients enable individualized testing of treatment approaches and studies into hereditary abnormalities. For example, modeling microcephaly using cerebral organoids has provided insights into how Zika virus infection affects neural development [13]. Similarly, intestinal organoids have been used to investigate the effectiveness of CFTR modulators on patient-specific mutations to explore cystic fibrosis [14].

Organoids offer a potent tool for toxic screening, drug development, and disease modeling. Instead of using traditional cell lines or animal models to predict medication responses, pharmaceutical companies increasingly use organoid-based platforms [15]. For instance, liver and kidney organoids have been used to evaluate drug-induced nephrotoxicity and hepatotoxicity, decreasing the need for animal testing and enhancing the early identification of side effects.

Developments in single-cell sequencing and multi-omics technology have further enhanced organoid-based disease research. By combining transcriptomics, epigenomics, proteomics, and metabolomics, scientists may analyze the molecular mechanisms underlying disease development at a level of detail never before possible. Tracking lineage trajectories throughout organoid development and finding uncommon cellular subpopulations have significantly benefited from single-cell RNA sequencing or siRNA-seq [16]. This has been essential for identifying treatment targets and comprehending tumor heterogeneity in cancer organoids.

The effectiveness of organoid technology has been shown in several significant molecular research. For example, lung organoids have been utilized to uncover host–pathogen interactions during SARS-CoV-2 infection [17], and colorectal cancer organoids have been used to identify novel treatment resistance mechanisms [18]. Organoid technology in cellular therapy is becoming more popular as it develops. Researchers are investigating organoid transplantation as a potential regenerative treatment for diseases like liver failure and retinal degeneration [19].

Despite their potential, organoids have several drawbacks. Their complete physiological significance is limited by the absence of immune system and vascular components, and inconsistent outcomes may arise from variations in differentiation techniques [20]. Ethical and regulatory issues further hamper clinical translation. However, continued efforts to combine vascularization, immune cell co-culture, and bioprinting technologies are anticipated to improve organoid complexity and applicability. In the future, standardizing organoid techniques, scaling production, and maximizing their therapeutic utility will require cooperation between basic researchers, physicians, and biotechnology businesses. With further advancements, organoid technology can transform drug development, regenerative therapy, and customized medicine, eventually leading to more potent cures for various illnesses.

While iPSCs have transformed regenerative medicine and disease modeling, their full potential can only be realized through precise genetic modifications. The integration of gene editing technologies, particularly CRISPR-Cas9, has opened new avenues for correcting genetic mutations, improving differentiation efficiency, and enhancing the safety of iPSC-derived therapies. CRISPR-based approaches enable targeted modifications with unprecedented accuracy, allowing researchers to rectify disease-causing mutations in patient-derived iPSCs before differentiation. This convergence of iPSC technology and genome editing enhances personalized medicine and accelerates the development of gene therapies for inherited disorders, cancers, and neurodegenerative diseases. The following section explores the evolution of CRISPR-Cas9, its applications in stem cell research, and the challenges in translating gene-edited iPSC therapies to clinical practice.

### Gene editing and CRISPR-Cas9

In molecular biology, gene editing has become a potent tool that allows precise DNA sequence adjustments to address genetic abnormalities, investigate disease causes, and develop new treatments. Because of its effectiveness, ease of use, and adaptability, CRISPR-Cas9 has transformed the field of gene editing among the different technologies. Since its discovery, CRISPR-Cas9 has been extensively used in fundamental and translational research, opening up new avenues for regenerative therapies and personalized medicine. However, despite its enormous potential, there are issues like off target impacts and moral dilemmas.

First discovered in bacteria, the CRISPR (Clustered Regularly Interspaced Short Palindromic Repeats) system is a natural defense against viral infections. Jinek et al. showed the potential of CRISPR-associated protein 9 (Cas9) as a programmable gene-editing tool [21]. A guide RNA (gRNA) is the component of this system that guides the Cas9 enzyme to a particular DNA region, where it causes a double-strand break. Then, cellular repair processes either use homology-directed repair (HDR) to enable precise editing or non-homologous end joining (NHEJ) to introduce mutations [22].

Compared to earlier gene-editing technologies like zinc-finger nucleases (ZFNs) and transcription activator-like effector nucleases (TALENs), CRISPR-Cas9 has several advantages. Because of its low cost, ease of design, and excellent efficiency, a variety of researchers can use it. Moreover, complex genetic modifications are made possible by its capacity to target multiple genes at once [23]. However, CRISPR-Cas9 also has drawbacks, such as the possibility of immune reactions to Cas9 in clinical settings, off-target mutations, and the low effectiveness of HDR-mediated repair [24].

Since its discovery, several improvements have improved CRISPR-Cas9 precision and versatility: high-fidelity Cas9 variants, like SpCas9-HF1, have been developed to reduce off-target effects [25]; newer technologies, such as base editing and prime editing, allow single-nucleotide changes without introducing double-strand breaks, improving safety for therapeutic applications [26]; and CRISPR interference (CRISPRi) and activation (CRISPRa) have been adapted for gene regulation instead of gene editing [27].

CRISPR-Cas9 has been employed extensively to model genetic illnesses, find pharmacological targets, and develop possible therapeutics. Preclinical research has shown its capacity to rectify genetic mutations in diseases like sickle cell anemia and Duchenne muscular dystrophy (DMD) [28, 29]. Patients have reported long-lasting increases in fetal hemoglobin levels in clinical trials employing CRISPR-edited hematopoietic stem cells for sickle cell disease and beta-thalassemia [30].

CRISPR-Cas9 has been instrumental in identifying genetic contributors to various diseases, including cancer and neurodegenerative disorders. Researchers have elucidated pathways in tumor growth and neurodegeneration by knocking out or modifying specific genes. Furthermore, CRISPR-based cell therapies, such as CAR-T cell engineering for cancer immunotherapy, have remarkably succeeded in clinical applications. Notwithstanding its achievements, CRISPR-Cas9 still faces several challenges. Developing more accurate genome-editing techniques is necessary because off-target consequences present dangers for therapeutic applications. Additionally, there are ethical questions, especially about germline editing and possible abuse of technology. The 2018 case of He Jiankui's illegal human embryo editing highlighted the need for stringent ethical standards and regulatory monitoring [31].

Developments in CRISPR-Cas9 technology are expanding the possibilities for regenerative medicine and gene therapy. Future studies will concentrate on increasing the range of genetic abnormalities that can be treated, improving delivery techniques, and improving specificity. While in vivo gene editing techniques may offer direct treatments for genetic illnesses, the combination of CRISPR and iPSCs holds promise for customized regenerative therapies [32]. To maximize CRISPR's therapeutic promise while maintaining ethical responsibility, interdisciplinary cooperation between researchers, doctors, and legislators will be essential as technology develops. In conclusion, gene editing has been revolutionized by CRISPR-Cas9, which offers a productive and adaptable instrument for biomedical research and treatment development. Even though there are still obstacles to overcome, revolutionary medical breakthroughs will be made possible by improving accuracy, delivery, and ethical supervision. CRISPR-based treatments could play a significant role in precision medicine as research advances, providing hope for patients with genetic disorders that were previously incurable.

Although CRISp-Cas9 presents excellent promise for fixing genetic diseases, its use in germline editing raises questions about accidental mutations, long-term effects, and the prospect of gene modification outside therapeutic need. Because of these concerns, many countries have set rigorous rules or complete prohibitions on germline genome editing. Before moving with clinical applications, the International Commission on the Clinical Use of Human Germline Genetically Editing and the World Health Organization (WHO) Expert Advisory Committee underlined the need for global oversight and ethical considerations (National Academies of Sciences, Engineering, and Medicine, 2020; WHO, 2021). Studies on the moratorium on germline editing also show a growing agreement that while somatic gene therapy develops, heritable genetic changes should be handled cautiously until safety, efficacy, and ethical questions are thoroughly addressed. Including various viewpoints helps us present a thorough analysis of the worldwide attitude on CRISp-Cas9 germline editing and its regulatory environment [33, 34].

## Stem cells in regenerative medicine

Rapid advancements in stem cell research have produced ground-breaking findings that have revolutionized our knowledge of disease models, cellular plasticity, and the development of new treatments. Novel applications in personalized medicine have been made possible by significant advancements in stem cell technology, such as improving iPSCs, single-cell multi-omics analysis, and gene-editing techniques like CRISPR-Cas9. These developments have given us strong tools for regenerative medicine and expanded our understanding of developmental biology. Translating stem cell research into clinical applications is becoming more and more possible as it advances, providing encouraging opportunities for organ regeneration, disease therapy, and tissue repair. The following section examines how regenerative medicine uses stem cells to tackle some of the most urgent issues facing the medical field.

### Neurodegenerative disorders (e.g., Parkinson’s, Alzheimer’s)

Because neurodegenerative diseases like Parkinson's disease (PD) and Alzheimer's disease (AD) grow over time and have no known cure, they pose a serious medical problem. A promising method for restoring damaged neurons, reducing neuroinflammation, and encouraging endogenous repair is stem cell treatment. Numerous preclinical and clinical investigations have examined the potential of different types of stem cells to treat neurodegenerative illnesses within the last 20 years. However, despite significant advancements, several issues still need to be resolved, including safety, effectiveness, and moral dilemmas.

Numerous preclinical investigations have shown that stem cells may be used to treat AD and PD. For example, dopaminergic neurons generated from ESC have been effectively transplanted into animal models of PD, improving motor function [35]. Similarly, iPSCs have demonstrated promise in AD models after differentiating into glutamatergic and cholinergic neurons [36].

**Fig. 3:**
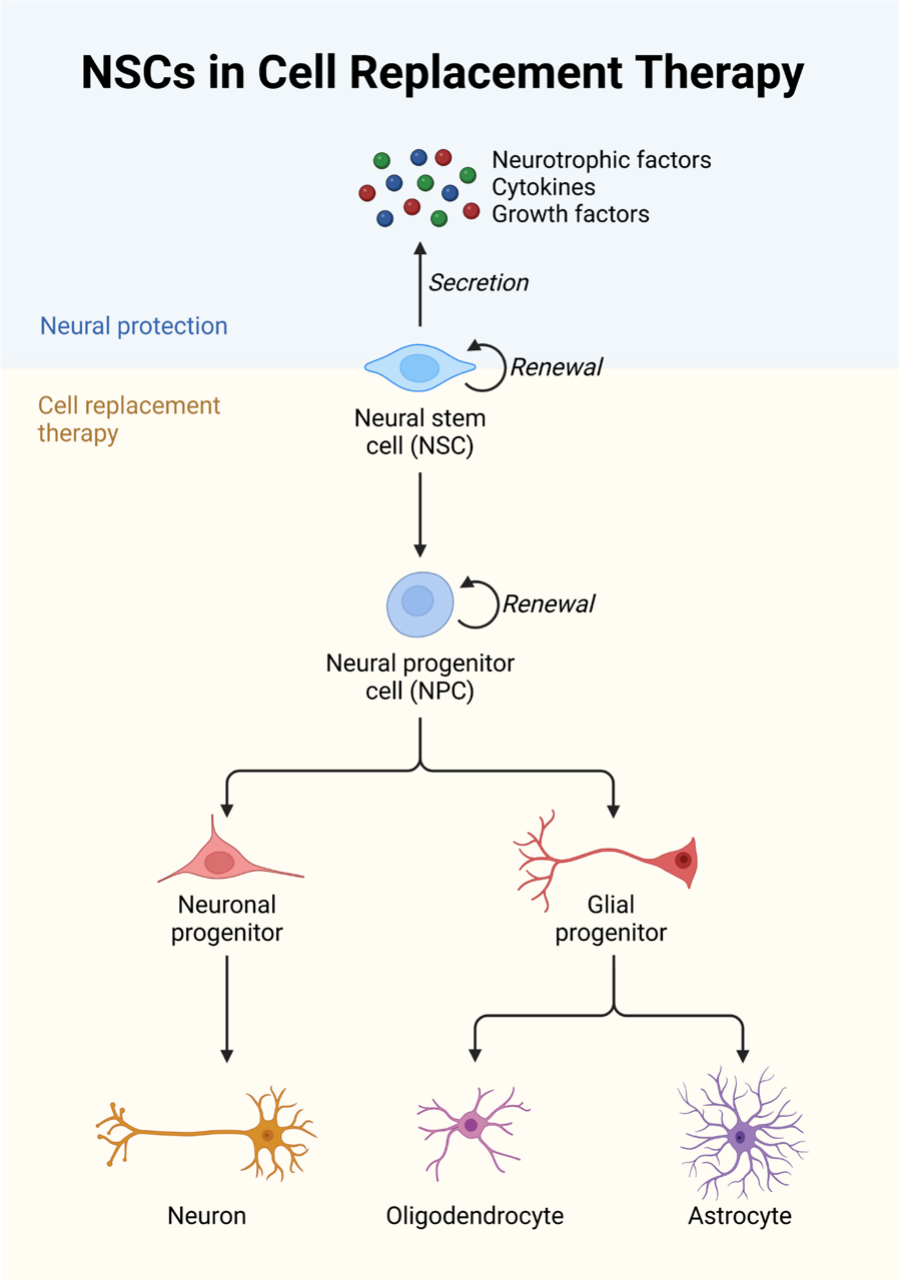
NSCs in cell replacement therapy. This picture depicts the function of neural stem cells (NSCs) in regenerative medicine, specifically in replacing lost or injured neural cells in disorders like stroke, spinal cord injuries, and neurodegenerative illnesses. It describes how NSCs can differentiate into neurons, astrocytes, and oligodendrocytes before being transplanted into impacted areas. The picture also highlights the possibility of NSC-based treatments for nervous system restoration

Recent developments in stem cell research have exposed the promise of human olfactory bulb-derived neural stem cells (hOBNSCs) for neurodegenerative diseases (Fig. 3). Marei et al. have exhaustively described the genetic and molecular characteristics of hOBNSCs, pointing out particular signaling pathways and epigenetic processes controlling cell proliferation, differentiation, and therapeutic applications. Their studies show that hOBNSCs might reduce cognitive deficiencies in Alzheimer's disease models and improve motor function recovery in Parkinson's disease models. By growing into functioning neurons, enhancing neuroprotection, and reducing neuroinflammation, hOBNSCs offer a viable path for cell-based treatments targeted at Alzheimer's and Parkinson's Disease. Dopaminergic neurons from the adult human substantia nigra and human embryonic neural stem cells were compared in Marei et al.'s thorough gene expression research. Agilent and Illumina Whole Human Genome Oligonucleotide Microarrays let the researchers identify distinct gene expression profiles in the two cell types. In hENSCs, genes linked to neurogenesis and cellular proliferation were elevated; genes related to neurotransmitter synthesis showed higher expression in DA neurons. These results provide significant fresh insights into the molecular characteristics of DA neurons and hENSCs, driving the development of stem cell-based treatments for neurodegenerative illnesses, including Parkinson's disease [37]. To better understand the different signaling pathways and epigenetic mechanisms controlling each cell type, Marei et al. (2012) compared gene expression analysis of human embryonic neural stem cells (hENSCs) and adult human olfactory bulb-derived neural stem cells (OBNSCs). With both cell types displaying activation of genes connected to neural progenitor proliferation, progenitor markers, and neural tube formation, their results indicated notable variations in transcriptional profiles. Of the 3,875 gene sets examined, 325 showed significant changes; pathway analysis revealed 75 sets, especially in the Notch, Wnt, and mTOR signaling pathways, which are essential for deciding neural stem cell destiny. Moreover, transcript variations linked with epigenetic changes demonstrated differing therapeutic potentials for hENSCs and OBNSCs concerning cell survival, proliferation, migration, and differentiation post-transplantation in central nervous system lesions. These results emphasize the need to know cell-specific molecular markers to improve stem cell-based treatments for neurological diseases [38]. Examining the possible therapeutic effects of hOBNSCs expressing human nerve growth factor (NGF), Marei et al. (2015) used a rat model of Alzheimer's disease (AD). Behavioral and memory tests showed that transplanting hNGF-expressing hOBNSCs significantly improved cognitive performance in rats with AD. The histological study of the hippocampal tissue found reduced amyloid-beta (Aβ) accumulation enhanced neuronal survival and synaptic plasticity. The transplanted cells lowered astrocytosis and microgliosis, promoting neurogenesis and control of inflammatory reactions. Based on their notable neuroprotective and neurorestoring properties, our studies imply that hNGF-expressing hOBNSCs are a reasonable substitute for cell-based therapy in Alzheimer's disease [39]. Human olfactory bulb-derived neural stem cells (hOBNSCs) were examined therapeutically by Marei et al. (2015) in a rat model of Parkinson's disease (PD). Behavioral assessments of the studies revealed that hOBNSC implantation significantly improved motor performance. According to histological and molecular studies, the transplanted cells raised striatal dopamine concentrations, enhanced neuronal vitality, and produced dopamine-like neurons. Reduced glial activation and neuroinflammation found in the study suggest that hOBNSCs help to neuroprotect and restore brain function. Based on the findings, hOBNSCs might be a cellular therapy for PD [40]. Emphasizing their contributions to neurogenesis and gliogenesis in nerve damage healing, Chen et al. (2024) address the important roles of FGF1 and IL12 in neuroregeneration. After nerve damage, fGF1 enhances neuroprotection and functional recovery by promoting neural stem cell (NSC) proliferation, differentiation, and survival. IL12 controls immune responses and neuroinflammation simultaneously, fostering a suitable condition for neuronal repair. By controlling these signaling channels, these elements have great potential to maximize therapeutic approaches meant to increase neuroregeneration and restore nervous system function [41].

Clinical studies have also shown motivating results. In Japan in 2018, a clinical study started using iPSC-derived dopaminergic neurons in PD patients (Takahashi, 2019). Assessed for their paracrine effects in controlling neuroinflammation in AD patients, phase I and II trials have shown possible cognitive benefits of MSCs. With some studies showing enhanced motor performance [42], transplantation of fetal-derived NSCs has been investigated as a treatment option for PD.

Stem cells enable neuroprotection and neuroregeneration by several means. These include direct cellular replacement, release of neurotrophic factors, and regulation of neuroinflammatory responses. For example, transplanted dopaminergic neurons can restore dopamine synthesis in PD, and MSCs and NSCs can secrete brain-derived neurotrophic factors (BDNF) and glial cell-derived neurotrophic factors (GDNF), which improve neuronal survival and synaptic plasticity in AD. Furthermore, stem cells reduce neuroinflammation by controlling microglial activity and preventing the release of pro-inflammatory cytokines.

One has many benefits from stem cell-based therapy. Unlike drug treatments, these can restore damaged neurons, lower neuroinflammation, and produce long-lasting results. Moreover, using patient-specific iPSCs helps enable customized treatments, reducing the possibility of immunological rejection. Still, some obstacles remain. Using ESCs continues to cause moral conundrums; iPSCs carry significant risks related to their capacity to cause cancer. Even after autologous transplantation, genetic instability resulting from reprogramming still presents problems with immune rejection. Stem cell therapies' requirement for perfect differentiation and integration into the host brain circuitry makes their practical deployment extremely difficult. Among the adverse effects of stem cell treatment in neurological illnesses are tumorigenesis, ectopic tissue development, and immunological rejection. According to Lindvall and Kokaia (2010) [43], transplanted cells are occasionally poorly integrated, which results in functional deficits instead of improvements. Moreover, several experimental experiments have shown that stem cells can unintentionally grow into non-neuronal cell types.

Usually, once iPSCs have gone through the reprogramming process, genomic stability is attained. Following reprogramming, the cells undergo adaptation and quality control when possible genomic changes, including those that can result in tumorigenicity, are extensively evaluated. Whole genome sequencing and other genomic studies, among other screening techniques, guarantee the safety of the iPSC-derived products. Notably, despite early worries about tumorigenic risks during reprogramming, no teratoma development or tumor growth has been documented in several clinical trials using iPSCs-derived products, supporting these cells' safety in regenerative medicine. This shows how well strict safety procedures reduce the residual dangers connected to iPSCs [8, 44].

Although immunological rejection is a significant factor in stem cell therapies, it is crucial to understand that this problem is not unique to those treatments. Any organ or cellular transplantation presents a frequent difficulty in immunological rejection, in which case the immune system may identify the transplanted cells or tissues as aliens and start an attack against them. Regarding stem cells, particularly those obtained from allogeneic sources, immune rejection is still a problem. However, several approaches are under investigation to lower these dangers, including immune suppression, the use of autologous cells, and the development of immune-evading cell treatments. The difficulties with immune rejection in stem cell treatment resemble those of conventional organ transplantation, emphasizing the requirement of thorough immunological control in both spheres of clinical activity [45, 46].

Combining methods are under investigation in many fields to improve the therapeutic effectiveness of stem cell treatment. Gene editing is an interesting approach whereby CRISPR-Cas9 technology fixes genetic mutations in iPSCs preparatory transplantation. This method has shown promise in slowing disease progression in preclinical animals by ensuring the transplanted cells are genetically modified for utility and viability. Another fantastic development is the concurrent release of BDNF and GDNF. These elements improve axonal development, help transplanted stem cells integrate into current brain circuits and enable neuronal survival. Moreover, immunomodulation has become important in increasing the effectiveness of stem cell treatment. Using MSCs engineered to secrete anti-inflammatory cytokines or combining stem cell transplantation with anti-inflammatory pharmacotherapy [47], researchers hope to improve the milieu for neuronal regeneration in diseases including AD and PD.

Finally, studies on biomaterial scaffolds to improve stem cell survival and integration within the cerebral milieu are in progress. Through better cell adhesion, controlled release of growth factors, and structural support, engineered hydrogels and biomaterial scaffolds improve the efficacy of stem cell treatments for neurodegenerative illnesses. Future studies should aim to improve post-transplant cell survival, lower cancer risk, and hone differentiation techniques. Safety and efficacy traits must be found through extensive clinical studies. Moreover, regulatory systems must be improved to help apply stem cell treatments in clinical settings. Because stem cells may replace damaged neurons and change disease etiology, they have great potential to cure neurodegenerative diseases. Notwithstanding significant advances, ethics, safety, and efficacy issues still need attention. Thanks to gene editing, biomaterial engineering, and combinatorial therapy developments, stem cell-derived medicines for AD and PD could proceed immediately.

### Cardiovascular renewal

**Fig. 4:**
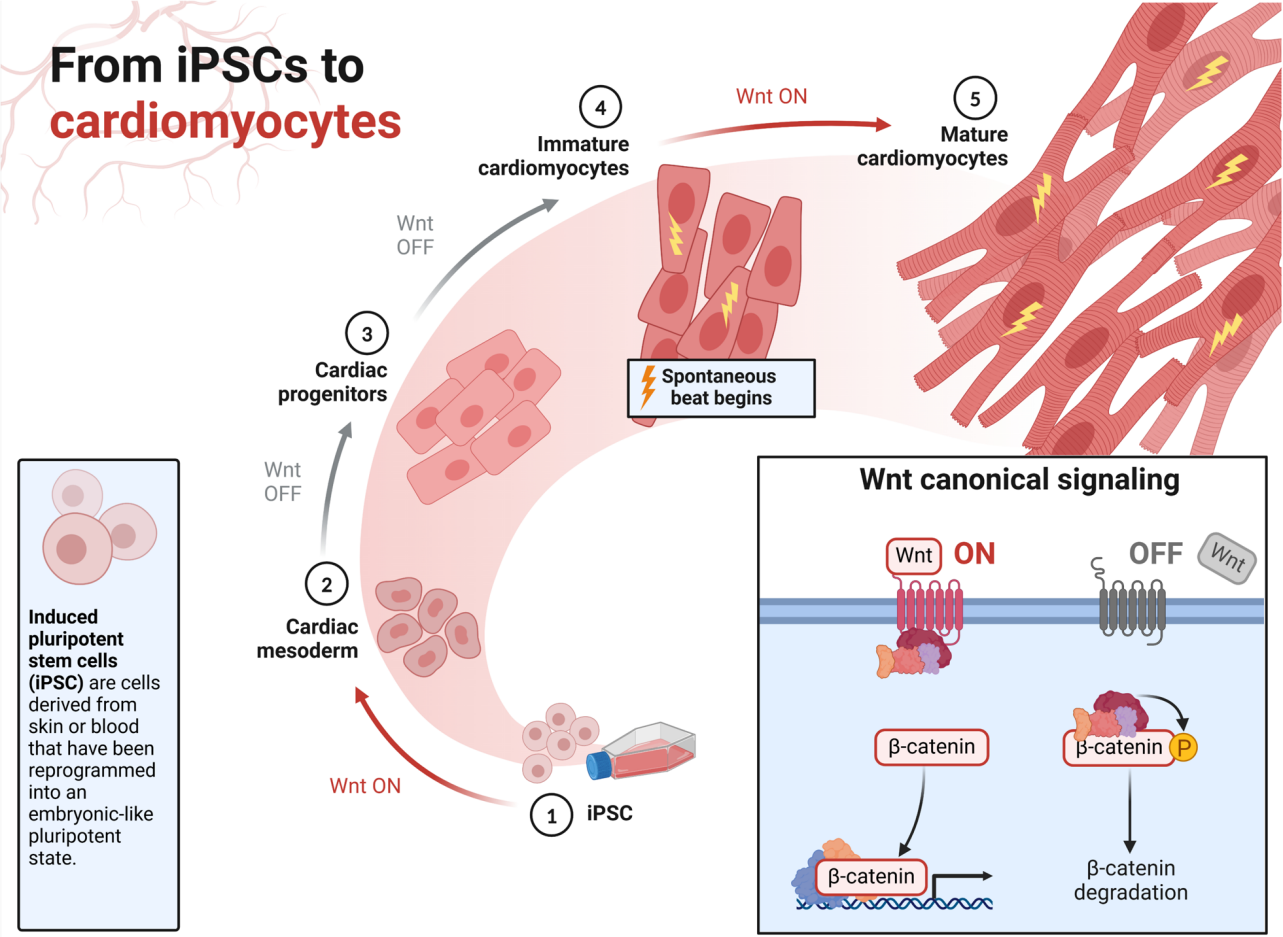
Wnt signaling during cardiomyocyte differentiation. This picture depicts in detail the role of the Wnt signaling system in controlling the differentiation of stem cells into cardiomyocytes. It demonstrates the temporal regulation necessary for practical cardiac lineage commitment by describing the dynamic involvement of Wnt activation and inhibition at various stages of heart cell development. The picture also shows important signaling pathways and molecular actors, showing how Wnt regulation can be used for cardiac tissue engineering and regenerative treatments

Still, the primary cause of illness and death worldwide is cardiovascular diseases (CVDs). The stem cell treatment is promising for repairing damaged cardiac tissue, enhancing cardiac performance, and slowing down the course of heart failure. Examined in cardiovascular regeneration has been the therapeutic potential of numerous stem cell types, including MSCs, cardiac progenitor cells (CPCs), iPSCs, and ESCs. Even with significant progress, improving stem cell survival, differentiation, and incorporation into host cardiac tissue remains difficult (Fig. 4).

Various kinds of stem cells have been looked at for heart repair. Their pluripotency and capacity to grow into functioning cardiomyocytes make ESCs and iPSCs perfect candidates for cardiac regeneration.

Direct differentiation, paracrine signaling, and immunomodulation help stem cells enable heart regeneration. MSCs and CPCs mostly rely on the paracrine secretion of growth factors such as insulin-like growth factor-1 (IGF-1), fibroblast growth factor (FGF), and vascular endothelial growth factor (VEGF) to exert their therapeutic effects. At the same time, ESCs and iPSCs can generate new cardiomyocytes. These elements help to lower fibrosis, improve neovascularization, and strengthen natural cardiac healing systems. Furthermore, by controlling inflammatory reactions, stem cells reduce oxidative stress and death in the heart affected by infarction [48].

Stem cell treatment has shown promise in helping heart function recover following a myocardial infarction. Left ventricular function, stimulation of neovascularization, and scar size reduction in rodent and large animal models have all improved following MSC, CPC, and iPSC-derived cardiomyocyte transplantation [49]. In a rat model, ESC-derived cardiomyocytes effectively integrated into the infarcted heart and promoted functional recovery, according to a 2007 study by Laflamme et al. Likewise, Silva et al. [50] showed in pig models of heart failure that human iPSC-derived cardiac cells enhanced cardiac function and lowered adverse remodeling.

Many clinical studies have assessed the safety and effectiveness of stem cell treatment in patients suffering from cardiovascular disorders. During the C-CURE trial, left ventricular ejection fraction in heart failure patients was shown to be improved by bone marrow-derived MSCs [51]. Promising safety results and functional improvement were revealed by the ESCORT study [52] evaluating the transplantation of ESC-derived cardiac progenitors in patients with severe heart failure. The ALLSTAR trial investigated myocardial infarction treatment using cardio-sphere-derived cells (CDCs); nevertheless, the results showed limited efficacy in extensive patient groups [53]. Although stem cell treatment shows great promise in these studies, different clinical results call for changes to cell delivery, survival, and integration techniques.

Stem cell treatment for cardiovascular regeneration has some restrictions and possible negative consequences, even if the outcomes seem encouraging. Tumorigenicity remains a significant problem with treatments derived from pluripotent stem cells [46], given the possibility of teratoma formation. Problems resulting from allogeneic transplantation and immunological rejection could perhaps restrict long-term efficacy. Moreover, inadequate engraftment and survival of transplanted cells lower therapeutic advantages, which calls for better cell transport methods [54]. Several clinical study subjects have shown arrhythmias, emphasizing the need for thorough safety evaluations before general clinical use [52].

To get beyond present constraints, researchers are looking for creative ways to improve the effectiveness of stem cell therapy. Gene editing techniques, including CRISPR-Cas9 [55], can help improve stem cells' vitality and uniqueness potential. Biomaterial scaffolds and 3D bioprinting [56] tissue engineering techniques are being developed to increase cell retention and integration. Furthermore, combining stem cell treatment with pharmacological drugs such as small-molecule inhibitors aiming at inflammation and fibrosis may improve the regeneration capacity [57]. Future studies should concentrate on standardizing cell production techniques and improving patient selection criteria to guarantee consistent therapeutic results. With the possibility of transforming the treatment of heart disease, stem cell treatment is a fascinating approach to cardiovascular regeneration. Cell survival, integration, and safety remain difficult even with encouraging findings from early-stage clinical studies and preclinical research. Potential approaches to improve the efficacy and clinical relevance of stem cell-based treatments are presented by gene editing, tissue engineering, and combination therapy developments. More research and cooperation are vital to maximize stem cell application for cardiovascular repair and enhance patient outcomes. Stem cell-based treatments seek to improve cardiac performance by encouraging myocardial repair and tissue regeneration. Examined for their capacity to differentiate into cardiomyocytes and other cardiac cell types, including endothelium and smooth muscle cells, are several stem cell types: ESCs, iPSCs, and MSCs. Early-phase clinical trials and preclinical studies on stem cell transplantation's improved heart function and myocardial healing [48, 58] have produced encouraging findings. Still, there are difficulties, including the low survival rate of transplanted cells, arrhythmias, and the necessity of best delivery techniques to produce long-lasting therapeutic results.

### Controlling diabetes

Stem cell research has lately advanced to provide exciting treatment opportunities for diabetes, especially Type 1 diabetes mellitus (T1DM). The several types of stem cells used in diabetes treatment, their mechanisms of action, advantages over conventional treatments, possible drawbacks or adverse effects, preclinical evidence for generating insulin-producing beta cells from iPSCs, clinical trials using stem cells for diabetes treatment, prospects in diabetes stem cell research, and recommendations for addressing current challenges are examined in this part. MSCs, iPSCs, and ESCs are being studied for diabetes treatment. Source from early-stage embryos, ESCs are pluripotent cells able to develop into any cell type, including beta cells releasing insulin. iPSCs reprogrammed from somatic cells to a pluripotent state—offer a patient-specific source for producing beta cells, reducing the danger of immunological rejection. MSCs have immunomodulating effects and are multipotent stromal cells able to develop into several cell types. Among the several organs they occupy are adipose tissue and bone marrow. MSC transplantation has been found to favorably affect both forms of diabetes mellitus without any noticeable side effects [59].

Stem cell treatments have various benefits over more traditional ones. The main advantage is the restoration of endogenous insulin generation, which lets transplanted beta cells react to blood glucose levels, enabling real-time glycemic control. This strategy could cut or replace the need for an exogenous insulin supply. Furthermore, long-term remission made possible by stem cell-based treatments could help to lower the risk of diabetes-related complications. According to a meta-analysis, MSC treatment could be helpful for both forms of diabetes mellitus with low side effects [60]. Furthermore, even if stem cell treatment shows promise, long-term safety and efficacy must be evaluated using large-scale clinical studies [59].

Preclinical research suggests that iPSCs can produce functioning beta cells. Differentiation techniques have been established to lead iPSCs through phases similar to pancreatic development, producing cells capable of secreting insulin in response to glucose. The effective reestablishment of normoglycemia by transplanting iPSC-derived beta cells in animal models points to their therapeutic effectiveness. Still, there are difficulties maintaining the effectiveness of differentiation and the continuous functionality of cells following transplantation [61].

Numerous clinical trials have studied stem cell-based treatments for diabetes. A Phase I/II randomized, double-blind, placebo-controlled investigation explored the application of allogeneic Wharton's jelly-derived MSCs (ProTrans) in individuals with newly diagnosed type 1 diabetes. The study reveals that beta cell activity may be maintained, and the medicine is considered safe [62]. In people with T1DM, Stem Cell Educator therapy has shown the potential to reverse autoimmunity and restore beta cell function [63].

Several categories demand further research to develop stem cell therapies for diabetes. Researchers can boost cell survival and function by finding strategies to augment transplanted cells' engraftment, survivability, and long-term efficacy. Based on comprehensive review and meta-analysis, stem cell treatment is a safe and successful therapy for people with diabetes mellitus. The study underlined the requirement of larger sample counts and longer follow-up times in subsequent studies [60]. Stem cell treatments can revolutionize diabetes by restoring endogenous insulin production and achieving continuous glycemic control. Despite much progress, more study is necessary to solve current problems and apply these drugs in clinical settings. More studies in this field could produce new treatments that improve the quality of life for people with diabetes.

### Transplantation of organ tissue engineering

**Fig. 5:**
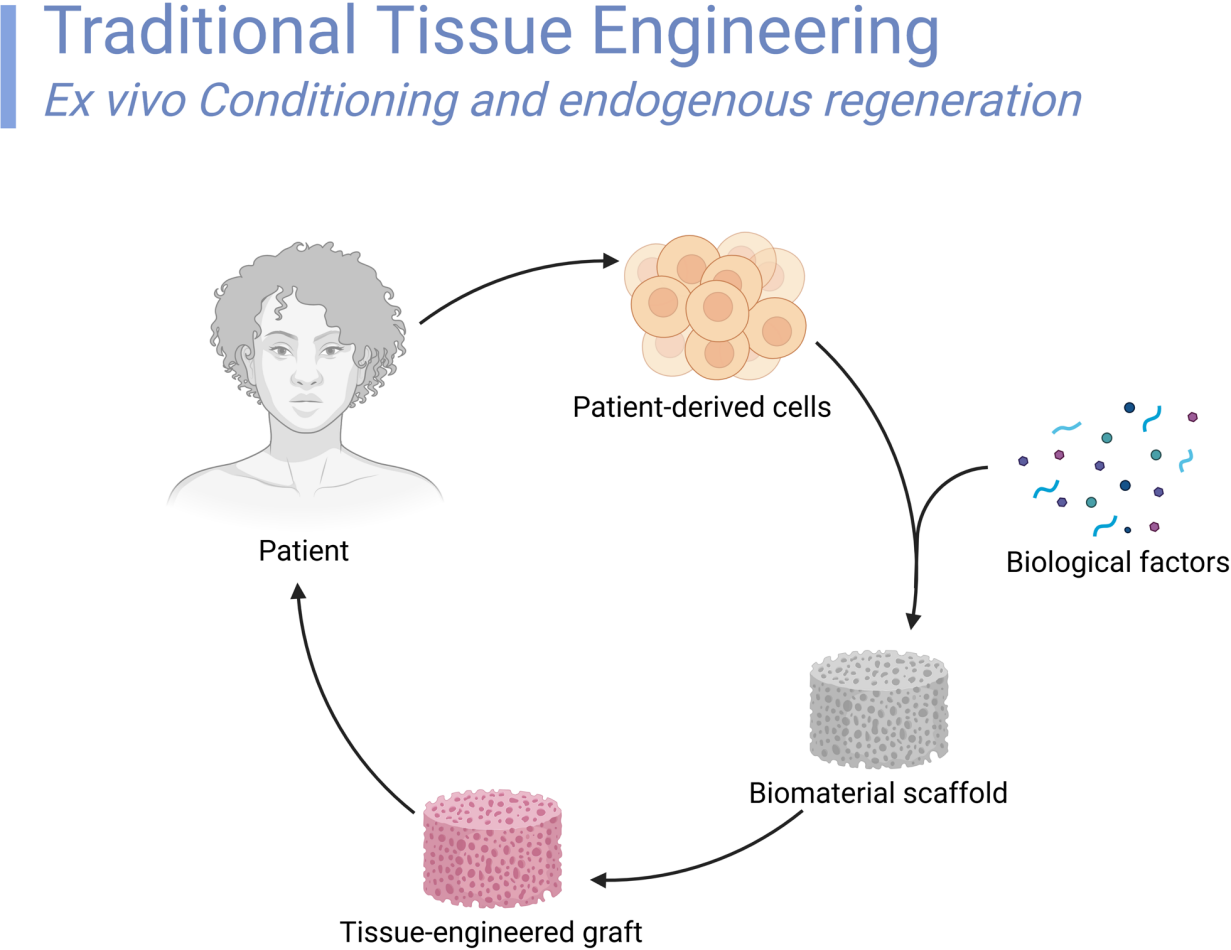
Traditional tissue engineering. This figure illustrates standard tissue engineering techniques, such as developing functional tissue constructs using biomaterial scaffolds, cell seeding, and bioreactors. It illustrates how cells are integrated into extracellular matrix-like scaffolding to support tissue integration and growth. The graphic also emphasizes the importance of biomaterials and bioactive chemicals in tissue development by highlighting important regenerative medicine applications such as skin grafts, bone regeneration, and vascular tissue engineering

Among the most interesting fields of regenerative medicine are stem cell-based organ transplantation and tissue engineering. Stem cells' ability to heal, replace, or repair damaged tissues and organs could help to meet the rising need for organ donations as well as offer treatments for some crippling diseases (Fig. 5).

This section examines modern stem cell utilization in tissue engineering and organ transplantation, including success stories, benefits, drawbacks, preclinical data, clinical results, and recommendations for future development. Among the most important achievements in stem cell-derived tissue engineering is the development of prosthetic skin for burn sufferers. In this regard, epidermal stem cells have produced successful skin transplants for patients suffering from severe burns. Stem cell skin grafts developed in clinical trials have been shown to hasten healing and greatly enhance patient outcomes [64]. Furthermore, those with skeletal traumas or osteoarthritis have benefited from carefully creating cartilage and osseous tissues using stem cells. These altered tissues have shown promise in both preclinical and clinical environments in reducing pain and restoring function. Studies have indicated that instead of conventional surgical techniques, MSCs can rebuild cartilage tissue.

In organ transplantation and tissue engineering, stem cells have several benefits. Because stem cells may differentiate into many cell types, they are essentially the perfect source for developing tissues and organs for transplantation. This feature helps develop patient-specific tissues from their cells, lowering the possibility of immunological rejection. Conventional organ transplantation also has a significant drawback in that stem cell treatments could drastically cut reliance on organ donors. Stem cells help injured tissues to regenerate, healing to be accelerated, and symptoms of degenerative disease to be reversed. A practical substitute for liver transplantation, patient-derived iPSCs can develop into functional hepatocytes for liver regeneration [65]. Moreover, continuous attempts to replicate whole organs' complicated structure and function complicate organ regeneration. In the lab, for instance, complex organs, including the kidney, liver, and heart, are much more challenging to grow than bioengineered tissues, including skin, cartilage, and lung tissue. The need for significant stem cell manipulation and culture aggravates issues about the cost-effectiveness and scalability of these therapies.

Preclinical research shows promises for developing functioning organs and tissues from stem cells. In animal models, successfully transplanting synthetic tissues, including skin, cartilage, and heart valves, has shown promise in restoring functioning and enhancing quality of life. Another success is the development of bioengineered lungs in a preclinical environment using stem cells to produce functionally active lung tissues that were efficiently implanted into animal models [66].

Stem cell-based tissue engineering is developing inside medicinal uses. One of the most remarkable examples is the use of MSCs in heart healing following myocardial infarction. According to clinical research, MSCs help cardiac tissue regeneration, enhance cardiac function, and lower the incidence of heart failure. Those who had heart attacks and underwent stem cell injections into their hearts showed notable improvements in their quality of life and cardiac function, according to a study written in the "Journal of the American College of Cardiology" [51].

Notwithstanding these therapeutic achievements, considerable obstacles remain to be overcome before the full promise of stem cells in tissue engineering and organ transplantation may be realized. The optimization of differentiation protocols for the development of functional tissues and organs is a crucial field that requires improvement. Even while stem cell differentiation into specialized cell types has advanced, these procedures still need to be improved to guarantee the creation of entirely functional tissues that blend in perfectly with the body. The growth of vascularization in bioengineered tissues is another significant obstacle. After being implanted, big tissues or organs cannot live without a functioning blood supply. To increase the survival and functioning of bioengineered tissues, researchers are investigating methods to integrate vascular networks into them. In conclusion, regenerative medicine has excellent promise in stem cell-based tissue engineering and organ transplantation. The success stories in tissue engineering for skin, cartilage, and heart demonstrate the therapeutic potential of stem cells in treating a range of illnesses. Despite the tremendous advancements, several obstacles remain, especially in scalability, organ complexity, and tumorigenicity. Future development in stem cell differentiation procedures, vascularization, and economic viability is essential if we overcome these obstacles and optimize the utility of stem cells in tissue engineering and organ transplantation.

## Ethical and legal challenges

Even if tissue engineering and stem cell-based organ transplantation have great promise, many ethical and legal issues must be resolved before these treatments are used broadly. These difficulties result from worries about the origins of stem cells, possible cancer hazards, and the need for consistent procedures to guarantee effectiveness and safety. Furthermore, regulatory systems are constantly changing to match the developments in this fast-growing research. Including stem cell-based treatments in clinical practice effectively depends on an understanding and ability to meet these ethical and legal obstacles. This change to address moral and legal issues emphasizes the need to define explicit rules to enable these discoveries' suitable and safe application.

### Ethical controversies and embryonic stem cells (ESCs)

**Fig. 6:**
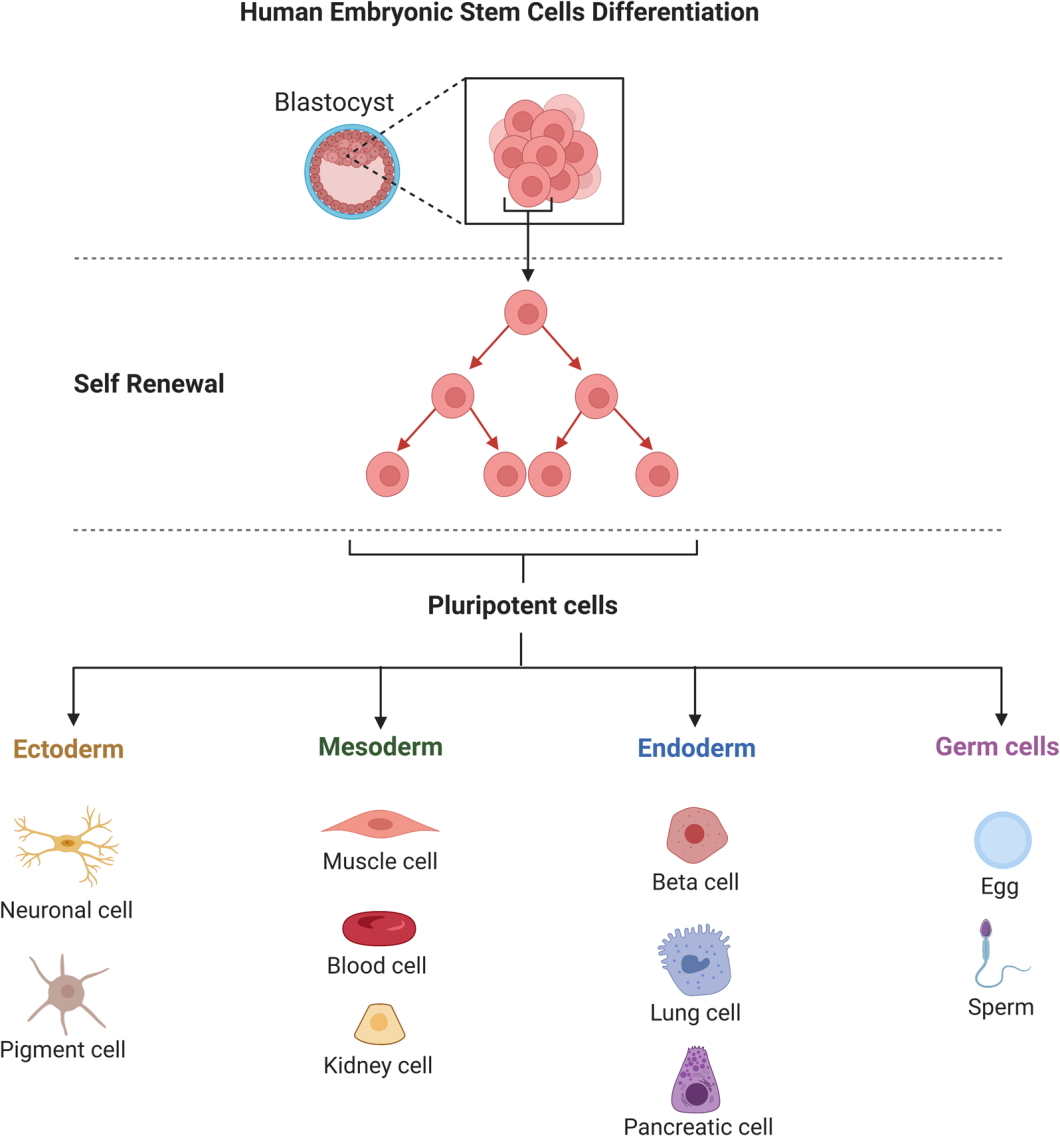
Human embryonic stem cell differentiation. This image shows the self-renewal process of human embryonic stem cells (hESCs) and how they differentiate into specialized cell types like muscle cells, blood cells, kidney cells, neuronal cells, pigment cells, ovum, and sperm. An outline of the molecular cues, including growth factors and transcriptional regulators, that direct lineage specification is given. Along with highlighting the potential of hESCs in regenerative medicine, the image also emphasizes the biological uses of differentiated cells, such as drug testing, disease modeling, and cell-based therapeutics

ESCs are pluripotent cells obtained from early-stage embryos. Because these cells can develop into almost all cell types found in the human body, they are essential for disease modeling, pharmacological research, and regenerative medicine (Fig. 6). Still, their approach to gathering begs serious ethical questions. Usually taken from blastocysts, embryonic stem cells cause the death of the embryo. This operation has spurred debates on the ethical consequences of destroying the embryo for scientific or medical progress as well as on its moral position. The main ethical concern related to the technique used to generate ESCs is that obtaining embryonic stem cells requires the destruction of a human embryo, a process some people find immoral. Many detractors of ESC research contend that human life starts at conception, so equating the death of an embryo with the act of killing a person. Many religious, philosophical, and ethical viewpoints hold that human embryos have inherent moral value and should not be used for research [67, 68]. On the other hand, supporters of ESC research contend that the possible advantages overwhelm any worries. They argue that, with just a few hundred cells and no mature fetus, blastocyst-stage embryos fall short in ethical terms compared to a fully developed human being. The great promise of ESCs in treating several diseases, including diabetes, heart disease, and PD, accentuates the ethical conundrum [69].

Issues about the control and supervision of ESC research, as well as the ethical consequences of embryo annihilation, remain unresolved. Although strict rules controlling the use of ESCs are in place in many nations, the execution of these rules varies greatly. The United States strictly controls the use of embryos for research and provides limited federal support for ESC research. Maintaining strict ethical standards, the UK has passed more lenient laws allowing the production of embryos, especially for scientific research. The differences in legislative systems highlight the ethical variety connected with ESC research and provide difficulties for international study. While those with limited standards may be worried about the ethical consequences of ESC research, nations with permissive rules could give great priority to significant scientific advancement and medical potential.

ESCs can transform regenerative medicine by producing organs and tissues for transplantation. Because they can transform into many specialized cell types—including neurons, cardiac cells, and pancreatic beta cells—ESCs are exciting options for treating diseases, including diabetes, cardiovascular disease, and spinal cord injury. Recent clinical studies show that cells obtained from ESC-derived cells could help treat macular degeneration, a major cause of blindness and heart failure following myocardial infarction. Successful implantation of ESCs-derived retinal pigment epithelial cells in first trials has improved eyesight in patients with age-related macular degeneration. Likewise, ESCs-derived cardiac cells have shown promise for patients with heart failure by displaying the capacity to rebuild damaged cardiac tissue [70]. Still, several ethical and practical issues limit the promise of ESCs in regenerative medicine. Lack of enough vascularization inside significant tissue constructs, which is necessary for feeding and oxygen delivery, adds still another obstacle to their effective transplantation [71].

To allay these issues, some scientists have looked at iPSCs as a substitute source of pluripotent stem cells. Without embryos, converting adult somatic cells into a pluripotent state produces iPSCs. This development has reduced ethical questions about ESCs. Still, iPSCs have certain drawbacks, mainly genetic stability and cancer. Though iPSCs have been used to develop cells for treating PD and other disorders, the long-term safety of iPSC-based therapies is yet unknown. Notwithstanding these problems, iPSCs offer a workable substitute for ESCs that could enable individualized treatment free from ethical questions about embryos [72]. Further study is required to guarantee their efficacy and safety, but other approaches, including iPSCs, present a potential path. As ESC research develops, reaching an equilibrium between tackling the ethical conundrums related to the use of these potent cells and advancing medical science will be very vital.

### Unregulated cellular clinics: deception, abuse, and the necessity of control

Stem cell treatment's promise to cure many ailments has attracted much interest worldwide. Unregulated stem cell clinics raise serious questions. For disorders including diabetes, spinal cord injuries, and PS, these clinics can offer untested and uncontrolled treatments. Though stem cell treatment has great promise, poor control, and supervision in these facilities results in fraud, exploitation, and major patient dangers. Marketing strategies, inadequate scientific data, and the exploitation of vulnerable people looking for alternative treatments for terminal diseases aggravate the problem of uncontrolled stem cell therapy. The main problems with unbridled stem cell clinics are exploitation and false application of stem cell treatments. These clinics often promote stem cell therapies as remedies for diseases lacking scientific support. In the lack of conclusive evidence on the effectiveness or safety of the treatments, they sometimes prey on patients' despair by promising false hope in exchange for significant financial gain. Sometimes, these clinics provide treatments using stem cells from questionable sources, such as unconfirmed adult stem cells or cells taken from animal tissues. National regulatory authorities with strict clinical trial and patient safety standards are often denied authorization for these drugs, such as the European Medicines Agency (EMA) and the U.S. Food and Drug Administration (FDA). So, many people turn to dangerous, dubious treatments that can have adverse effects [73, 74].

Many significant drawbacks follow from the absence of control for stem cell treatments offered by unregistered clinics. These cover the possibility of infections, tumor development, strong allergic reactions, and problems resulting from stem cell injections. The lack of standardization in stem cell preparations is a big problem that might cause patients to have different dosages, cell kinds, or cell cultures whose safety has not been sufficiently evaluated. People who had stem cell injections from unapproved clinics, for example, have complained of acquiring tumors or infections. Many Americans who got unlicensed stem cell therapies for macular degeneration at an unapproved Florida facility in 2014 were found to have an uncommon type of eye cancer. Another instance relates to a Mexican facility where patients received injections of illegal stem cells taken from aborted fetal tissue. Significant negative consequences, including tumor growth, autoimmune reactions, and mortality, followed from this [75].

Improved rules and supervision are required to stop the spread of unregistered stem cell clinics. International organizations and national health authorities must impose severe rules prohibiting running unapproved stem cell centers. The first step is to improve the openness of stem cell treatments by mandating all stem cell-based therapies to undergo thorough preclinical and clinical testing with data available to the public and medical community. Regulatory authorities, including the FDA in the United States, have to step up their search for and close bogus clinics distributing illegal drugs. Furthermore, patients and medical professionals must be appropriately informed about unauthorized stem cell treatment hazards. Public knowledge-enhancing initiatives help to reduce demand for some services and direct consumers toward safe, evidence-based substitutes [76].

Between 700 and 1,000 uncontrolled stem cell clinics are thought to be operating worldwide, mostly in nations with insufficient control, including the United States, Mexico, and Thailand, according to the International Society for Stem Cell Research (ISSCR, 2019). These clinics' promise of quick access to treatment draws patients from nations with strict laws most of the time. Law enforcement and control suffer from the worldwide character of the problem. Patients may fly overseas to receive illegal treatments, even in nations with tight regulations on stem cell therapies, therefore confounding attempts to guarantee their safety. Several nations have addressed the issue. The FDA warned numerous U.S. clinics about offering unapproved stem cell treatments in 2017; in 2019, it started legal action against a Florida clinic for the illegal marketing of stem cell-based products (U.S. Food and Drug Administration, 2019) [77]. Likewise, clinics providing unlicensed stem cell treatments in Japan have paid hefty fines to the government since these treatments are closely monitored. Nonetheless, such initiatives have to be global in scope and entail international collaboration to offset specific institutions' unethical behaviors.

A good way to increase the effectiveness of stem cell treatment going forward is the development of global regulatory criteria that supports the evolution and application of safe stem cell therapies. A more coherent global framework for stem cell control will give scientists and medical practitioners an enhanced means to guarantee stem cell therapies' safety, reliability, and efficacy. Reducing bogus clinics' impact also helps speed the licensing process for stem cell treatments. As research in stem cell biology and technology develops to guarantee the safety and efficacy of stem cell-based treatments, the discipline must emphasize enhancing cell purity, lowering tumorigenic potential, and refining procedures. Strong laws and enforcement, along with international cooperation, are vital to prevent the use of stem cell technologies for dubious and dangerous treatments.

Critical studies from organizations, including the World Health Organization (WHO) and the U.S. Food and Drug Administration (FDA), have included the risk of untested stem cell treatments being provided in clinics without sufficient control. Among the several stem cells under discussion are hESCs, iPSCs, and MSCs. To protect public health and guarantee scientific integrity in clinical applications, this version offers a more transparent and evidence-based debate on the need for stricter control and monitoring of stem cell treatments [76].

In summary, patients run significant hazards from unbridled stem cell clinics due to exploitation, dishonesty, and lack of control over stem cell treatments. Many times, these clinics use dangerous, dubious, and maybe harmful treatments to target vulnerable people. To control stem cell research and eradicate dishonest clinics from operation to solve this problem, global regulatory authorities have to apply strict laws and rules. These rules must be strictly followed to safeguard patients and guarantee the safe execution of stem cell treatments. Stem cell treatments' development depends on ongoing research and development funding. Hence, international cooperation is essential to ensure these treatments fully realize their promise safely and successfully.

### Reproducibility and standardizing

Stem cell treatments must be fully realized by addressing reproducibility issues and standardizing them to reach their promise. The relevance of these issues in stem cell research is discussed in this part, with analyses of the elements affecting repeatability and suggestions for improving the standardizing of stem cell treatments. Stem cell therapy presents an excellent promise in treating many ailments, including neurological conditions, diabetes, cardiovascular problems, and some types of cancer. Challenges related to reproducibility and standardization have hampered the progress of stem cell-based treatments from the laboratory to clinical application; these are fundamental causes of the ongoing difficulties in obtaining consistent and reliable results across different laboratories and clinical settings.

Successful development of stem cell treatments depends on the basic scientific research idea of reproducibility. Reproducibility in research is the capacity of another researcher to carry out the same experiment under identical conditions and get the same results. Reproducibility is crucial in stem cell research since even small changes in cell management or experimental technique can produce notable effects on the outcomes. In stem cell research, reproducibility problems could result from several causes, including unequal cell sourcing, inconsistent culture conditions, and variations in the molecular characterization of stem cells [68, 78]. Thus, the advancement of stem cell-based treatments depends critically on consistent techniques that enable homogeneity between laboratories and research. Little changes in temperature, pH, oxygen levels, and nutrient concentrations will affect stem cells' proliferation, differentiation potential, and genetic integrity since they are highly susceptible to their milieu. This variability could cause differences in the characteristics of stem cells derived from different sources, influencing the therapeutic uses and experimental results. Obtained from many tissue sources, MSCs could have various degrees of differentiation potential, immunological properties, and therapeutic efficacy [79]. This is the cause of the lack of repeatability in stem cell research. The culture techniques will affect the pluripotency and differentiation capacity of iPSCs and ESCs. Improving repeatability calls for precise stem cell description and validation in line with accepted procedures and standardization of cell culture settings. One further obstacle to repeatability is the absence of consistent procedures for stem cell development. Because they may develop into various cell types, stem cells, especially iPSCs, have great promise for regenerative therapy. Often complicated, the present differentiation techniques might not be repeatable in multiple labs. Differentiating iPSCs into dopamine-producing neurons for PD treatment or insulin-producing beta cells for diabetes control depends on the exact control of culture conditions, growth factors, and signaling pathways. Variations in these elements could affect the therapeutic potential of distinct cell populations, therefore affecting the efficacy and purity of the populations. Sometimes, these approaches are not globally standardized, which results in inconsistent findings from different studies [80]. To improve repeatability, researchers must set and follow proven, tailored, standardized differentiation protocols for every cell type.

Standardizing stem cell therapy is as vital as repetition to guarantee the safety and efficiency of stem cell-based treatments. Establishing globally established rules and procedures for developing, characterizing, and clinical using stem cell products forms part of the standardizing process. The safety and efficacy of stem cell therapies depend on standardized stem cell isolation, proliferation, differentiation, and quality assurance techniques. Stem cells must be free of toxins, genetic abnormalities, and the possibility of cancer before they are used for therapy. Regulatory authorities, including the FDA and EMA, have formulated recommendations for stem cell-based therapy to provide uniform procedures. These recommendations are now under development and call for further cooperation among academics, doctors, and regulatory bodies to offer complete guidelines capable of general adoption [81].

The lack of consistent manufacturing techniques is a significant obstacle to the broad clinical implementation of stem cell therapy. Generating clinical grade iPSCs and their derivatives for regenerative medicine depends on strict environmental control for cell growth and development. Any variation from these guidelines could produce cells that are unfit for clinical application. Researchers and doctors must build strong manufacturing techniques (GMP) for stem cell production, follow strict quality control procedures, and guarantee that stem cell-based treatments satisfy necessary safety and efficacy criteria, thereby addressing this obstacle [82].

Developing Good Manufacturing Practices (GMP) for stem cell therapies ensures these treatments' safety, consistency, and reproducibility in clinical settings. The FDA CFR 21 regulations are essential in standardizing cell-based therapies, including cardiological applications, by providing guidelines for manufacturing, testing, and distributing stem cell products (U.S. FDA, 2020). Adherence to these guidelines is vital for minimizing the risks associated with stem cell therapies, such as tumorigenicity and immune rejection. Furthermore, the potential for personalized approaches using iPSCs derived from patients' cells offers a promising strategy for overcoming immune rejection and optimizing treatment outcomes. Ongoing research into the molecular and cellular mechanisms of cardiac repair and integrating stem cell therapy with other regenerative strategies is expected to lead to more effective and widely applicable treatments for cardiovascular diseases [82].

Standardized procedures and repeatable approaches will help to validate stem cell therapies in clinical trials. The inability to compare data among several research groups resulting from discrepancies in preclinical trials may cause stem cell-based medicines to move from laboratory environments to clinical applications to be delayed. Standardized approaches will enable improved preclinical research and help to guarantee that clinical studies rely on exact, repeatable data. Moreover, regulatory authorities will be more equipped to assess their safety and efficacy if stem cell treatments are grounded on accepted methods and strict testing processes [83].

In essence, it is necessary to solve the issues of repeatability and homogeneity to exploit stem cell-based treatments effectively. Differences may hamper stem cell research's repeatability in manufacturing techniques, differentiation methods, and cell culture conditions, postponing the valuable application of therapies. Together with strong manufacturing standards for stem cell-based therapeutics, standardized methods for stem cell growth, differentiation, and characterization must be developed to improve reproducibility. Establishing thorough standards for stem cell treatment's safety, efficacy, and consistency depends on cooperation among researchers, doctors, and regulatory authorities. The advancement of stem cell-based therapies to clinical application and safe, efficient treatment provision for patients depends on resolving these challenges.

### Issues about long-term safety and effectiveness

Standardization and repeatability are essential for stem cell therapy to achieve consistent clinical results and regulatory approval. Still, despite well-defined procedures and manufacturing guidelines, the long-term safety and effectiveness of stem cell-based treatments remain significant questions. Notwithstanding the encouraging findings of the first clinical studies, concerns about possible hazards like tumorigenicity, immunological rejection, and accidental differentiation must be sufficiently addressed. Effective integration of stem cell treatments into conventional medicine depends on preclinical research and prolonged clinical follow-ups, which evaluate their long-term benefits. The next part examines the primary safety and efficiency issues that must be resolved to guarantee patients' consistent and continuous use of stem cell treatments.

A possible treatment for various diseases, including degenerative diseases and autoimmune disorders, is stem cell treatment. Early-stage clinical studies show positive outcomes. However, questions about these medicines' long-term safety and effectiveness still exist. Many issues have to be resolved if stem cell treatments are to be successfully included in standard medical treatment. Stem cell treatment's longevity of therapeutic effects is a central issue. Based on extensive patient data showing an acute myocardial infarction [84], stem cell therapy may enhance left ventricular ejection fraction (LVEF) for as long as three years post-treatment. Comparably, studies on lentiviral gene therapy for Wiskott-Aldrich syndrome have revealed long-lasting clinical improvement. Accurate assessment of the lifetime of therapeutic effects depends on prolonged follow-up in clinical trials since not all treatments have long-lasting results; some drugs show only temporary effects.

A significant issue is still the safety of stem cell treatment. Even though many studies show good safety profiles, possible hazards, including carcinogenesis, immunological responses, and therapy failure, must be considered. Although MSC therapy has been mostly well tolerated, questions about possible long-term side effects, including the possibility of cancer, still exist.

Following accepted therapeutic guidelines is vital since uncontrolled stem cell treatments have had adverse effects. There are serious questions regarding the spread of unapproved treatment that unlicensed stem cell clinics provide. These behaviors compromise patient safety and erode public confidence in credible scientific projects. Strict rules imposed by regulatory authorities help to stop the improper application of stem cell treatments. Ethically, medicines should be grounded on strong scientific evidence, and patients should be informed about possible hazards and benefits.

### Biotechnology and market expansion: investments

Stem cell therapy has become a transforming tool in regenerative medicine, providing possible treatments for various disorders, including degenerative diseases and major injuries. This active industry has drawn significant investments and is seeing a notable market expansion. The present situation of biotech investments in stem cell therapy and the factors driving market growth are examined in this part. The global stem cell therapy market has grown significantly throughout the past ten years. At USD 11 billion in 2022, the market is expected to rise at a compound annual growth rate (CAGR) of 14.8%. By 2032, it will reach USD 44 billion. The main drivers of this growth are the growing demand for regenerative medicines, developments in stem cell research, and sound regulatory systems. Rising from USD 14.15 billion in 2023 to around USD 48.89 billion by 2033 [85], the US stem cell therapy industry is expected to grow at a compound annual growth rate (CAGR) of 13.2%.

Key variables driving this growth are more clinical studies, more financing for stem cell research, and the building of GMP-certified manufacturing plants. Stem cell treatment's growing promise has attracted significant investments in government agencies, pharmaceutical corporations, and venture capitalists. Venture capital investment in stem cell research and development peaked in 2024, with a sizable portion going to businesses concentrated on creative stem cell uses. This flood of money has helped develop new drugs and hastened the translation of clinical applications from laboratory research. D mergers and acquisitions (M&A) have significantly changed the scene of stem cell treatment. Big pharmaceutical firms aggressively purchase biotech startups or form relationships to diversify their regenerative medicine portfolios. Many well-known acquisitions in 2024 demonstrated how strategically important stem cell technology is in filling inefficiencies in medicine.

Many important reasons are driving the expansion of the stem cell treatment market. Improved knowledge of stem cell biology, made possible by ongoing research, has helped develop more targeted and potent treatments. Governments all around are passing laws supporting the progress of stem cell research and treatments through funding projects and quicker approval procedures. The growing prevalence of degenerative and chronic diseases has driven demand for creative treatments and positioned stem cell therapy as a reasonable substitute. Growing knowledge of the possible advantages of stem cell treatments has resulted in higher patient acceptance and interest, hence driving more market demand.

Despite its optimistic growth trajectory, the stem cell therapy sector faces hurdles, including rigorous laws, costly treatment development expenses, and ethical problems. Public confidence building and regulatory approval acquisition depend on thorough clinical research confirming the safety and effectiveness of medications. Strategic alliances, technical developments, and a growing emphasis on individualized therapy are expected to drive even further business expansion. As science advances and more drugs get approved, stem cell treatments should become more available, giving hope to those with once-incurable diseases. Supported by significant investments, the stem cell therapy company is pioneering medical innovation. As the market develops, cooperation among researchers, doctors, investors, and legislators will be crucial to overcome obstacles and actualize the potential of stem cell therapies in changing healthcare.

### Anticipation against actuality: from the societal viewpoint

The rapid development in stem cell therapy has attracted significant biotech investment and driven market expansion, suggesting that regenerative medicine shows a bright future path for healthcare. Thanks to more support from both public and commercial sectors, stem cell-based treatments are becoming increasingly commercialized and give hope to those with once-terminal diseases. Still, public opinion of stem cell treatment is often shaped by scientific studies, media coverage, and unfounded assertions independent of this excitement. Differentiating the real promise of stem cell treatments from the exaggerated expectations that could lead to misunderstandings as research advances is crucial. This difference between public opinion and scientific progress shapes the present debate on stem cell treatment and underlines the need for open communication and reasonable expectations.

With the possibility of treating several serious diseases, stem cell treatment has attracted significant attention as a potential development in regenerative medicine. A complex interaction of media coverage, ethical debates, and scientific advancement often shapes public opinion of stem cell treatment. This study shape’s public opinion and media representation by contrasting the inflated assertions about stem cell treatments with the current scientific reality, guiding expectations and impressions. The media dramatically shapes public opinion of medical developments, mainly stem cell treatment. Studies show that the media presents a too-positive view of the therapeutic use of stem cell research, which could cause exaggerated hopes about the availability and speed of new treatments. Published in Science Translational Medicine [86], much of the research emphasizes possible breakthroughs while insufficiently addressing the related scientific and legal limitations.

Stem cell clinical trial participants share their stories on social media, enhancing this impact. These first-hand stories could provide insightful analysis; nevertheless, they could also unintentionally compromise scientific credibility, create unreasonable expectations, and violate confidentiality. Researchers have noted these problems, and they also advise rules controlling social media posts on stem cell clinical experiments [87].

Media framing, political ideas, ethical issues, and media points of view all help to define public opinion about stem cell research. Studies show that people's opinions on stem cell research could be influenced by their surroundings and the questions asked. Results of the Gallup poll show different opinions on government financing for stem cell research, usually defined by political and ethical differences. Ethical debates have often split public opinion, especially around research on ESCs. Media coverage can periodically enhance this polarization by presenting false viewpoints, overstretching claimed benefits, or stressing primarily moral criticism. Such depictions might cause uncertainty and the development of false ideas [86].

Though stem cell research has great promise, much time and work are needed to translate laboratory findings into workable treatments. Managing regulatory protocols, addressing ethical issues, and running thorough clinical studies to validate therapy, safety, and efficacy constitute challenges. The public may be misled concerning the availability and readiness of stem cell treatments by the media's inclination to spread discoveries without enough background information.

Concerns have been raised by the rise of uncontrolled facilities offering experimental stem cell treatments. These clinics regularly take advantage of public enthusiasm by offering treatments lacking scientific support, eroding confidence in reliable research, and seriously compromising patient health in the debate on stem cell treatment, facts, and hype conflict. Although stem cell research has great promise, media representations should show fair and truthful facts. Educating the public on scientific approaches, especially the challenges and deadlines related to creating new treatments, will help bring expectations into line with reality. Encouragement of informed public debate helps stakeholders to guarantee that hope stems from scientific knowledge and supports the ethical evolution of stem cell treatments.

### Access, affordability, and medical inequalities

As stem cell treatments progress and get regulatory approval, questions about their cost and accessibility are becoming more acute. Many patients suffer despite the transforming potential of these sophisticated medical therapies because of their high cost, limited availability, and complicated legal systems. Societal events, geographical differences, and healthcare infrastructure all help to magnify inequalities in the capacity to profit from these findings. These issues must be solved if stem cell treatments are broadly available and provide fair healthcare solutions for all instead of being limited to wealthier communities.

Emerging as a promising front line in medical research, stem cell therapies can treat many diseases. Still, major issues, though, the cost and availability of these drugs aggravate healthcare inequalities. The financial obstacles patients encounter, the difficulties obtaining stem cell treatment, and the broader consequences for healthcare equity are investigated in this section. Variables impacting the availability of stem cell treatment include geographic location, healthcare infrastructure, and socioeconomic level. Patients undergoing specialist treatment may need access to facilities that are not in every area. Furthermore, limiting the availability of stem cell treatments could affect the capacity of healthcare facilities and the knowledge of medical personnel. Sometimes, these limitations cause patients to be excluded from possible treatment alternatives or result in long waiting times.

Many people find great difficulty with the significant cost of stem cell treatments. Sometimes, these treatments require sophisticated equipment and complex procedures, significantly increasing costs. For many, these stem cell injections might cost $16,500 per joint—an unacceptably high amount. Different policies exclude experimental or unapproved treatments; insurance coverage for stem cell therapy is inconsistent. Patients are driven to look for other financial solutions, including personal loans or crowdsourcing, to pay for the operations. Financial obligations might cause patients great stress, which might cause them to stop treatment altogether.

### Exorbitant pricing and limited access interact to aggravate already existent healthcare disparities

Socioeconomic elements, including job level, educational background, and income level, can influence a person's access to and funding capacity for stem cell treatments. Studies show that differences in access to treatments like HCT generally correspond with socioeconomic level, complicating treatment availability in underdeveloped areas. Furthermore, common outside of stem cell treatment is healthcare inequities. Several approaches could be suggested to help to solve these problems. Enacting rules that support fair access to stem cell treatments, including insurance coverage and help for underprivileged areas, will help to reduce inequalities. Patients can seek suitable treatment if the public is informed about the availability and advantages of stem cell treatments. Moreover, funding studies to create reasonably priced stem cell treatments might improve the availability and economy of treatments. Stem cell treatments have great promise, but it is essential to face issues with accessibility, cost, and healthcare inequalities. By putting in place thorough laws, society can ensure that everyone from all walks of life or socioeconomic levels may access stem cell medicine.

### Potential risks and ethical considerations in stem cell therapy

Stem cell treatment presents several challenges, even if it has great possibilities for regenerative medicine. The excitement for its medical possibilities has to be balanced with ethical considerations and knowledge of likely negative consequences. Three main questions—tumorigenicity, immunological responses, and ethical concerns—are investigated in this part. A significant concern in stem cell treatment is cancer. Comprising ESCs and iPSCs, pluripotent stem cells (PSCs) can differentiate into any cell type. Nevertheless, their self-renewing characteristics raise the risk of teratoma development if differentiation is not well controlled. During in vitro proliferation, adult stem cells, including MSCs and NSCs—can undergo genetic and epigenetic changes leading to malignant transformation. Researchers have looked at several approaches to lower undifferentiated cell populations, genetic and epigenetic screening to identify and destroy aberrant clones, and the use of suicide gene systems, such as inducible caspase-9, to eliminate proliferating cells following transplantation specifically [88, 89].

Immunological reactions generated by stem cell-based treatments could cause rejection, inflammation, or transplant failure. Usually avoiding immunological rejection, autologous stem cell treatment—using self-derived cells—may cause inflammatory reactions. On the other hand, allogeneic stem cell treatment—using donor-derived cells—has a higher risk of immunological rejection and calls for immunosuppressive medication. Though patient-specific, iPSC-derived cells may retain immunogenic markers depending on insufficient reprogramming or epigenetic memory [90]. Modern approaches include gene editing techniques like CRISPR-Cas9 to remove immunogenic surface proteins, the development of hypoimmunogenic stem cell lines by changing major histocompatibility complex (MHC) expression, and the advancement of universal donor stem cells via new immune-evasive engineering [91].

**Fig. 7:**
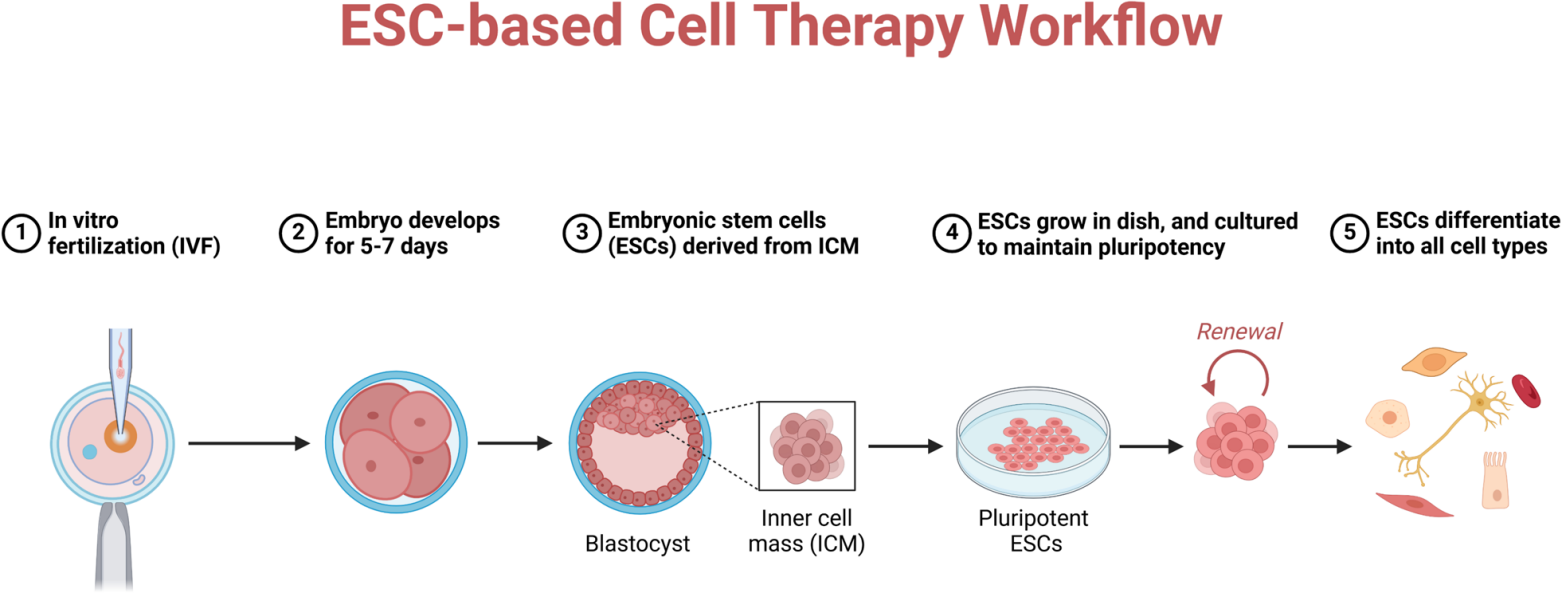
ESC-based cell therapy workflow. This figure shows a detailed procedure for using embryonic stem cells (ESCs) in cell-based treatment. It discusses ESC separation, quality control procedures, their regulated development into functional cell types, and final patient transplantation. The picture illustrates how ESC-derived cells treat degenerative diseases, repair damaged tissues, and promote customized medicine while highlighting important factors, including immunological compatibility, safety concerns, and therapeutic efficacy

Particularly about the use of ESCs, which require the destruction of human embryos, stem cell research and therapy create serious ethical conundrums (Fig. 7). This is still a divisive bioethical issue, and several nations have developed strict policies. Using CRISPR-Cas9 in germline editing raises a significant ethical conundrum since it might cause unexpected genetic changes with inheritable consequences. While somatic gene editing is becoming more and more approved, ethical and safety concerns typically rule against germline therapies [92]Furthermore, hurdles related to regulations and marketing persist since early support of dubious stem cell treatments has led to false claims and patient mistreatment. Protecting patient safety and preserving scientific integrity depend on strict clinical validation and regulatory measures [93].

Including stem cell treatment in mainstream medicine requires careful risk assessment and ethical debate. Tumorigenicity, immunological reactions, and ethical questions must be addressed as research advances to improve safety procedures for the responsible evolution of stem cell treatments. This study guarantees a complete evaluation of the transforming power, and the inherent risks connected to stem cell treatment, thereby supporting the material in the title, a revolutionary cure or a pandora's box.

## Future perspectives

Future views in stem cell therapy have primary goals addressing safety, efficacy, and accessibility issues and ensuring ethical behavior and regulatory conformance. Using gene editing technologies to improve stem cell therapy, safety, and effectiveness is a significant development. More focused and powerful treatments result from CRISPR-Cas9, which improves stem cell functioning or fixes genetic flaws. Gene editing in stem cell treatment could help create customized treatments catered to particular people, enhancing outcomes and reducing dangers. Furthermore, gene editing guarantees the regulated behavior of stem cells, lowering cancer risk and other consequences. The future of stem cell treatment depends critically on the development of consistent clinical procedures. By reducing treatment variability, standardization will guarantee consistent results across several patient populations and research environments. Establishing procedures for procuring, processing, and transporting stem cells can help guarantee consistent results and support more general clinical applications. By strengthening regulatory control, one may also guarantee that medicines are safe and efficient before they launch into the market. Maintaining good treatment quality and building industry confidence as more stem cell-based treatments become clinical trials depending on standardized methods. Stem cell treatment progress calls for strengthening ethical and legal systems. As these treatments get more accessible, especially with patient consent and stem cell procurement—ensuring respect for ethical norms become increasingly important. Regulatory authorities have to keep improving their control to meet the unique needs of stem cell treatments and keep a balance between patient safety and creativity. Clear moral values and stricter laws will help to prevent exploitation and abuse as well as provide a basis for the moral development of stem cell treatment. In the end, improving the availability of stem cell treatments will be essential for fulfilling their full potential. As demand for these treatments increases, answering questions about availability, cost, and geographical inequalities is imperative. If steps are taken to lower their exorbitant costs, especially by improving manufacturing techniques and extending insurance coverage, more patients will have access to stem cell treatments. Furthermore, improving healthcare infrastructure would guarantee that, especially in underprivileged areas, patients from many backgrounds can gain from developments in stem cell treatment. By improving accessibility and fairness in stem cell treatment, we can guarantee that these new medications are reachable to people most in need.

## Conclusion

To sum up, stem cell research shows great promise as a creative solution for many diseases and injuries, possibly providing treatments for once-impossible problems. While the development of standard clinical procedures is intended to improve the quality and homogeneity of treatment, advances in stem cell therapy—primarily through gene editing—have resulted in more focused and effective therapies. Still, some challenges arise even with these encouraging developments. The control of illegal stem cell clinics, the ethical conundrums related to embryonic stem cell use, and the availability and cost of medications constitute significant issues to solve. The future of stem cell treatments depends on cooperative efforts to improve regulatory frameworks, guarantee fair access to therapy, and boost safety and efficacy through creative technology. Overcoming these obstacles as we progress depends on public, legislative, and scientific community cooperation. To balance patient safety and innovation, regulatory authorities must continuously improve their monitoring systems, creating an environment that supports development. Shaping opinions of stem cell treatments, guaranteeing informed decision-making, and lowering the frequency of unlicensed clinics depend primarily on public education and awareness campaigns. Ultimately, stem cell treatments can transform medicine, yet their effectiveness will rely on the responsible and fair application of technical developments. Therefore, it is imperative to investigate the benefits and drawbacks of this fast-changing issue.

## Data Availability

The paper and its supplementary information contain all the data supporting this review article.
